# Calculation of exit dose for conformal and dynamically‐wedged fields, based on water‐equivalent path length measured with an amorphous silicon electronic portal imaging device

**DOI:** 10.1120/jacmp.v12i3.3439

**Published:** 2011-03-03

**Authors:** Awusi Kavuma, Martin Glegg, Mohamed Metwaly, Garry Currie, Alex Elliott

**Affiliations:** ^1^ Department of Clinical Physics and Bioengineering Radiotherapy Physics Beatson West of Scotland Cancer Centre Glasgow G12 0YN Scotland UK; ^2^ College of Medicine, Veterinary and Life Sciences, Department of Clinical Physics University of Glasgow Glasgow G12 8QQ Scotland UK

**Keywords:** transit *in vivo* dosimetry, EPID, exit dose, equivalent path length (EPL)

## Abstract

In this study, we use the quadratic calibration method (QCM), in which an EPID image is converted into a matrix of equivalent path lengths (EPLs) and, therefore, exit doses, so as to model doses in conformal and enhanced dynamic wedge (EDW) fields. The QCM involves acquiring series of EPID images at a reference field size for different thicknesses of homogeneous solid water blocks. From these, a set of coefficients is established that is used to compute the EPL of any other irradiated material. To determine the EPL, the irradiated area must be known in order to establish the appropriate scatter correction. A method was devised for the automatic calculation of areas from the EPID image that facilitated the calculation of EPL for any field and exit dose. For EDW fields, the fitting coefficients were modified by utilizing the linac manufacturer's golden segmented treatment tables (GSTT) methodology and MU fraction model. The nonlinear response of the EPL with lower monitor units (MUs) was investigated and slight modification of the algorithm performed to account for this. The method permits 2D dose distributions at the exit of phantom or patient to be generated by relating the EPL with an appropriate depth dose table. The results indicate that the inclusion of MU correction improved the EPL determination. The irradiated field areas can be accurately determined from EPID images to within ± 1% uncertainty. Cross‐plane profiles and 2D dose distributions of EPID predicted doses were compared with those calculated with the Eclipse treatment planning system (TPS) and those measured directly with MapCHECK 2 device. Comparison of the 2D EPID dose maps to those from TPS and MapCHECK shows that more than 90% of all points passed the gamma index acceptance criteria of 3% dose difference and 3 mm distance to agreement (DTA), for both conformal and EDW study cases. We conclude that the EPID QCM is an accurate and convenient method for *in vivo* dosimetry and may, therefore, complement existing techniques.

PACS numbers: 87.50.ct, 87.55 D

## I. INTRODUCTION

Patient dose verification on a daily basis is becoming increasingly important in external‐beam radiotherapy, and measurement of dose in the patient at the time of treatment (*in vivo* dosimetry) is widely accepted as an important part of the quality assurance (QA) program to validate the accuracy of the planned and delivered dose. Many recent studies have highlighted the capabilities/advantages of EPID for this purpose, in addition to positional verification and linac QA.^(^ ^1^ ^,^ ^2^ ^,^ ^3^ ^)^

Kairn et al.^(4)^ first suggested the possibility of radiotherapy treatment verification using radiological thickness by the quadratic calibration method (QCM) of an EPID. Using the same QCM approach, Kavuma et al.^(5)^ reported on the prediction of exit dose based on water‐equivalent path length (EPL) measured with an amorphous silicon EPID for open fields with the Varian portal imager. The relationship between intensity and phantom thickness (T) for a pixel at location (i,j) in the detector is assumed to be quadratic function of thickness, given by:

(1) $$A(i,j)*T^{2}(i,j)+B(i,j)*T(i,j)+\ln\left(\frac{M\;(i,j)}{M_{o}(i,j)}\right)=0$$

where *A(i,j)* and *B(i,j)* are quadratic coefficients related to the attenuating coefficients of the materials; $M_{o}(i,j)$ is the integrated image signal obtained without any material in the beam; *M(i,j)* is the EPID signal obtained after imaging solid water phantom of thickness *T*. By acquiring several EPID images of solid water phantoms of thicknesses in range 0–35 cm and fitting the acquired data to Eq. (1), the solutions of *A(i,j)* and *B(i,j)* are obtained by least square methods.

The quadratic solution of Eq. (1), given by

(2) $$x_{n}(i,j)=\frac{-B(i,j)\pm \sqrt{B^{2}(i,j)-4A(i,j)\ln[M_{1}(i,j)/M_{o}(i,j)]}}{2A(i,j)}$$

is the approximate (initial solution) equivalent path length for any other material imaged by the detector resulting into signal $M_{1}(i,j)$.^(^ ^4^ ^–^ ^5^ ^)^ The equivalent path length is the converged solution $x_{n+1}(i,j)$, reached iteratively according to Eq. (3) below:^(5)^

(3) $$EPL(i,j)=x_{n+1}(i,j)=\frac{A(i,j)x_{n}{\left(i,j\right)}^{2}-c(i,j)}{2A(i,j)x_{n}(i,j)+B(i,j)}where\;c(i,j)=\ln[CF_{s,z}(i,j)\left(\frac{M_{1}(i,j)}{M_{0}(i,j)}\right)]$$

and ${\text{CF}}_{s,z}(i,j)$ is the scatter correction factor if a patient/phantom is imaged at any other irradiation situation other than the reference condition — that is, the signal $M_{1}(i,j)$ has to be corrected for field size, phantom scatter, and monitor unit changes. Kavuma et al.^(5)^ proposed a method for conversion of EPL to dose using percentage exit thickness dose (PETD), a parameter derived from the tissue phantom ratio (TPR), where the exit dose map for open fields were computed from

(4) $$D_{ext}(i,j)=MUxOFxB(s)x\frac{PETD(i,j)}{100}xE_{p}(r,z)xB_{p}(x,z,s)$$

where *MU* is the given monitor unit; *OF* is the output factor of field *s* relative to the $10\times 10\;{\text{cm}}^{2};B(s)$ is a factor which corrects for reduced backscatter proximal to the exit surface (corrects dose in our setup to reference conditions); $E_{p}(r,z)$ and $B_{p}(x,z,s)$ are the envelope and boundary profiles, respectively, used to correct for the flood‐field effect of flattening the beam profile.^(^ ^5^ ^–^ ^6^ ^)^ The exit dose is defined at the dose plane positioned at a distance $d_{\text{max}}$, from the exit surface of the phantom/patient,^(7)^ where $d_{\text{max}}$ is equal to the depth of dose maximum. It should be noted that the exit dose does not refer to the exit surface dose.

In the present work, we report the feasibility of using the method in other clinical radiotherapy treatment situations, by comparing exit doses predicted with EPID and treatment planning system (TPS) for irregular and enhanced dynamic wedge fields. To obtain additional independent verification of EPID calculated exit doses, MapCHECK 2 (SUN Nuclear Corporation, Melbourne Florida, USA) was calibrated and used to measure directly the exit doses for solid water phantoms.

Radiation fields that are not square, rectangular or circular are termed as irregular fields. An irregular field also has an equivalent square field that will yield the same value of a given dose function.^(8)^ The dose functions of interest are the scatter correction factor, ${\text{CF}}_{s,z}(i,j)$, that depends on irradiated area and thickness, and the output factor (*OF*) that depends on irradiated area as described above in Eqs. (3) and (4), respectively. Enhanced dynamic wedge dose profiles are computer controlled, created by sweeping one of the Y jaw collimators from starting (open field) to closed position (0.5 cm from the opposite fixed Y jaw), while both the X jaws remain fixed during irradiation.^(^ ^9^ ^–^ ^10^ ^)^ Because of the collimator motion, different parts of the field are exposed to the primary beam for different lengths of time, creating a wedged dose gradient across the field — hence, the coefficients A(i,j), B(i,j) and $M_{o}(i,j)$ used for open fields may not be appropriate. Another set of coefficients was generated by using the golden segmented treatment table (GSTT) for 60° wedge angle and open beam data that were used for EDW radiation fields.

Commercial transit EPID modules, where radiation doses can be verified in the presence of patient permitting routine *in vivo* dosimetry, are unavailable. The purpose of this study is to use the QCM algorithm to model irregularly‐shaped and dynamically‐wedged fields. In addition, the effect of the EPID nonlinear response at low monitor unit (MU) on EPL will be incorporated into the algorithm. These improvements are designed to increase the accuracy and applicability of the algorithm in routine patient *in vivo* dosimetry.

## II. MATERIALS AND METHODS

### A. Treatment unit and EPID detector

A Varian linear accelerator (Clinac iX series; Varian Medical Systems, Palo Alto, CA) with a 120 leaf millennium multileaf collimator (MLC), an Exact‐Arm imager support, and an a‐Si‐500 EPID (hardware/software IDU‐20/IAS3) was used in this study. The investigations were limited to 6 MV photons and a dose rate of 400 MU/min unless otherwise stated. The imager was positioned at a fixed source‐to‐detector distance (SDD) of 140 cm, but the results were scaled to source‐to‐isocenter distance (SID) of 100 cm for comparisons. All fields were defined at SID. Dark field and flood field calibration of the imager were carried out according to the vendor's recommendation, to obtain a flat and noiseless image. The treatments were scheduled in the ARIA record and verify system (Varian Medical Systems) and accelerator operated in the clinical mode. The acquired images were accessed from ARIA via the Varian portal dosimetry software and exported into MATLAB v2008b (The MathWorks, Inc., Natick, MA) as ASCII files for further analysis. Further details on setup, image acquisitions, EPL, and exit dose calculations are given in a previous studies.^(^ ^4^ ^–^ ^5^ ^)^

### B. Monitor unit effects on equivalent path length

Preliminary results demonstrated significant dependence of calculated EPL on delivered monitor units, more especially at lower values. The effect of varying MU and the influence it has on the calculated EPL was studied by irradiating solid water materials of thicknesses 10, 20 and 32 cm for square field sizes of 5, 10 and 20 cm; at 20, 50, 100, 200, 300 and 500 MU; and dose rates of 400 MU/min and 100 MU/min. The average EPID response (signal) in a 13×13 pixel (approximately 1.0 cm^2^ area) region of interest in the center of the image was generated for each of the three thicknesses and field sizes. A dosimetric parameter, the signal‐to‐monitor‐units ratio (SMUR), was calculated by dividing the EPID signal by the delivered number of monitor units. The main purpose of the SMUR calculation is to take into consideration the nonlinear response of EPID at lower MU. The data were normalized to those at 100 MU. From these results, correction factors dependent on the MU were determined and Eq. (2) above was modified to take into consideration the effect of MU on EPL. Hence, the overall correction due to phantom thickness, field area, and monitor unit is given by:

(5) $$CF_{s,z,mu}(i,j)=\frac{CF_{s,z}(i,j)}{CF_{mu}}$$

This correction factor ${\text{CF}}_{s,z,\text{mu}}(i,j)$ becomes the coefficient of the $M_{1}(i,j)/M_{0}(i,j)$ term in Eq. (2).

### C. Equivalent path length and exit dose for irregular (conformal plain) fields

**Figure 1:**
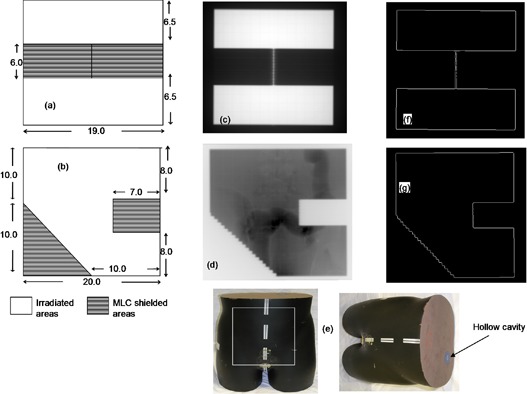
Figures (a) and (b) show the irregular‐shaped apertures of varying shapes and areas; dimensions are in cm measured at the isocenter. Figure (c) shows EPID image obtained after radiating the 15 cm solid water and MapCHECK device using field (a). Figure (d) shows the EPID image obtained after irradiating the anthropomorphic phantom (Fig. (e)) using field (b). Figures (f) and (g) show their respective boundaries extracted from the irradiated images.

As mentioned above, the field area and resulting phantom scatter are essential for predicting the EPL from an EPID response. In the case of irregular fields, one method of obtaining the area is to determine it from the EPID image. A range of irregular‐shaped fields of known field area were designed using the Eclipse (Varian Medical Systems, Palo Alto, California, USA) TPS. The field sizes were defined by the MLC leaves with the collimator jaws opening remaining fixed at $25\times 25\;{\text{cm}}^{2}$. To verify the accuracy of calculated equivalent path length and resulting dose, corresponding doses predicted with the TPS were compared with doses calculated from EPID images. MapCHECK 2, a 2D radiation detector, was calibrated to measure exit dose as an additional dose variation method. According to the manufacturer, MapCHECK 2 is a 2D array of 1527 uniformly spaced diodes, an active detector resolution area of 0.64 mm^2^, a diode– diode spacing of 7.07 mm, capable of covering an area of 32×26 cm at the isocenter. The device has a buildup and backscatter to the sensitive region of 2.0±0.1 and $2.75\pm 0.1\;{g/\text{cm}}^{2}$, respectively. The MapCHECK device was first calibrated by positioning it on the couch and aligning its detector level with the isocenter. It was then irradiated with a direct anterior (gantry angle zero) beam of 100 MU to a $10\times 10\;{\text{cm}}^{2}$ field size. The software allows the entrance of a specified factor (dose corresponding to a given number of MU at specific depth and field size), which is used to normalize the data to create a calibration file that was used to correct the dose maps for all subsequent measurements. To avoid heavy weight above the MapCHECK device, it was positioned upside down on top of 15 cm thickness of homogeneous solid water (total water equivalent material of MapCHECK and solid water is approximately/equal to 20 cm). The gantry was rotated to 180°, such that the sensitive side of MapCHECK device faced the beam direction. The MapCHECK and solid water were CT scanned and images were exported to TPS. (Figure 1a) shows the MLC shape used for the MapCHECK/EPID detectors measurements. The plan was exported to the Linac and 100 MU exposure taken, subsequently acquiring EPID image (Fig. 1(c) and MapCHECK measurements at the same time. In addition, an anthropomorphic lower torso phantom shown in Fig. 1(e) (The Phantom Laboratory, Salem, New York, USA) was positioned on the treatment couch and an anterior MLC shaped field of Fig. 1(b) was taken with 200 MU. The phantom consists of natural‐human‐skeleton lumber vertebra, pelvis, upper third of femur, and a hollow cavity (reproduces the sigmoid flexure and rectum) cast into Urethane (material with same effective atomic number as the body soft tissue). The corresponding image acquired by EPID is shown in Fig. 1(d). A MATLAB code was written to read and detect the radiation field edges from the EPID acquired images. The EPID image was first converted into a binary image. The edge detection algorithm is based on searching the entire EPID image and calculating the approximate gradients of the image intensity function. An edge is localized at those points where the gradients are maxima. The image pixels identified as forming edges are set to one with all other pixels being set to zero. By tracing the entire bounded region (irradiated area), edge polygons are formed, as shown in (Figs. 1f) and (g).

The coordinates of the pixels of these polygons are searched and successively stored. If the X and Y coordinates of all vertices are known and entered in order of successive vertices, then the area of the polygon^(11)^ at the isocenter can be calculated using the equation:

(6) $$Area=\frac{1}{2}\left(\left(x_{1}+x_{2})(y_{1}-y_{2})+(x_{2}+x_{3})(y_{2}-y_{3})+\ldots +(x_{n}+x_{1})(y_{n}-y_{1}\right)\right)\left(\frac{pix}{mag}\right)$$

where *n* is the total number of vertices, $x_{i}$ and $y_{i}\;;i=1,2,\ldots ,n$ are the x and y coordinates of each edge pixels making the bound region; *pix* is the pixel size (resolution) = 0.784 mm and *mag* is the magnification. The sides of the equivalent square areas are obtained from taking the square roots of the area in Eq. (6). The area calculation was also tested with other images acquired for square, rectangular, and wedged fields. The calculated areas using the equation above were compared with the expected area and percentage differences computed.

The calculated area is used to establish an appropriate scatter factor CF(i,j), which is used, together with the correction coefficients A(i,j), B(i,j) and the image signal obtained without any material in the beam $M_{o}(i,j)$ for open fields, to predict the EPL as described in Eq. (3). The same area is used to establish an appropriate output factor (OF), which is found by interpolation between output data table points for square field sizes. The exit dose is then calculated, as described in Eq. (4), where the PETD due to thickness z and field area s is related to TPR according to the following equation:^(5)^

(7) $$PETD(z,s)={\left(\frac{SAD}{SAD+z/2-d_{\max}}\right)}^{2}TPR_{ef\;f}(z,s)$$

where *SAD* is the source‐to‐axis distance, $d_{\text{max}}$ is the maximum depth, and ${\text{TPR}}_{\text{eff}}$ is the TPR at an effective distance $z-d_{\text{max}}$. The calculated EPL beneath the MLC shielded regions (approximately/ equal to 100 cm) is higher than the maximum depth at which TPRs have been measured for the linac. PETD is used in the conversion of 2D EPL map to exit dose map. To facilitate the calculation of doses in these regions, the TPR table was extrapolated from 40 cm to 50 cm. For all calculated EPL greater than 50 cm, the TPR was set to the MLC transmission factor, where the average MLC transmission is defined as EPID dose (signal) ratio at the central axis of a closed MLC measurement to an open beam, for the same field size of $10\times 10\;{\text{cm}}^{2}$.^(12)^ That is

(8) $$MLC\_Transimision=\frac{MLC\_Closed\_EPID\_Signal}{Open\_Field\_EPID\_Signal}=0.013$$

To compare the dose distributions from the MapCHECK device (sensitive region is 2.75 cm) with the EPID exit dose (calculated at 1.5 cm), the doses from the former were dosimetrically adjusted. The MapCHECK dose map was resampled to the same number of 2D data points as those used for the EPID and TPS. The effect of resampling the MapCHECK data points was initially investigated and found to have insignificant adverse effect on the 2D dose map. Similarly all the TPS 2D dose data were extracted 1.5 cm from the phantom exit. All doses for EPID, MapCHECK, and TPS are absolute, measured in cGy.

### D. Enhanced dynamic fields

There are two potential approaches for verification of dose for EDW, either using the same set of correction coefficients A(i,j), B(i,j) and $M_{o}(i,j)$ generated for open fields, or generating another set of coefficients that involve the wedge motion in the field. Our investigations revealed that the former approach has some limitations in predicting EPL, especially for EDW angles above 30° (see Section IV Discussion, below) and, as a result, in this study we used the latter approach.

All measurements were performed using the Y1‐IN EDW orientation. The wedged image signal without any material in the beam $M_{0}^{\theta }(i,j)$ was calculated for the largest symmetric EDW field size of $20\times 20\;{\text{cm}}^{2}$ (used in this study) with moving jaw Y1=10 cm, fixed jaw Y2=10 cm, and fixed length X=20 cm, using the MU fraction calculation methodology with GSTT.^(^ ^10^ ^,^ ^13^ ^)^ For simplicity, the GSTT was represented analytically as an exponential function:

(9) $$GSTT(Y)=a_{0}+a_{1}\exp(b_{1}Y)$$

where *Y* is the moving jaw position that ranges from −20 to 10 cm; the fitting coefficients $a_{0},a_{1}$ and $b_{1}$ are determined from the Varian published values.^(14)^ According to the MU fraction model,^(^ ^9^ ^,^ ^13^ ^)^ the full‐field segmented treatment table associated with wedge angle θ is then given by:

(10) $$STT_{\theta }^{F}(Y)=MU\left(\frac{w_{\theta }GSTT(Y)+w_{0}GSTT(0)}{w_{\theta }GSTT(Y=9.5)+w_{0}GSTT(0)}\right)/k$$

where *MU* is the applied monitor units; *GSTT(0)* and GSTT(Y=9.5) are the GSTT at Y=0 and Y=9.5, respectively (9.5 is due to the fact that the Varian wedge stops 0.5 cm from the fixed jaw position); *K* is a geometrical correction factor that scales the data from the isocenter to the EPID imager level,

(11) $$w_{\theta }=\frac{\tan(\theta )}{\tan(60)}\;and\;w_{0}=1-w_{\theta }$$

The STT is a two‐entry table composed of 20 segments (21 instances), which gives the positions of the moving jaw versus the proportion of the delivered monitor units for each field segment. To simplify data manipulation with the EPID, which is comprised of 384×512 pixels, the following steps were taken. First linear interpolation of the ${\text{STT}}_{\theta }^{F}(Y)$ table was used to create 384 points (in wedge direction), where all the points outside the desired field length are reduced to zeros. Secondly this row of 384 pixels is replicated 512 times to constitute matrix $M_{0}^{\theta }(i,j)$.

To establish the appropriate coefficients for the wedged fields, an additional measurement and subsequent derivation of $A_{w60}(i,j)$ and $B_{w60}(i,j)$ for the 60° wedge was made. The correction coefficients for any other wedged angle θ $\left(A_{w\theta }(i,j\right)$ and $B_{w\theta }(i,j)$) were obtained from those of the 60° wedge and the open field $\left(A_{w0}(i,j\right)$ and $B_{w0}(i,j)$), using weighting factors obtained by the ratio of tangents in a way analogous to that applied to the GSTT.^(9)^ That is

(12) $$A_{w\theta }(i,j)=(w_{\theta })A_{w60}(i,j)+(w_{0})A_{w0}(i,j)$$

(13) $$B_{w\theta }(i,j)=(w_{\theta })B_{w60}(i,j)+(w_{0})B_{w0}(i,j)$$

With all the necessary factors established, the EPL for the irradiated material can be calculated as described in Eq. (3) above. The EPL calculated in this way is as if the wedge does not exist.

The exit dose can then be calculated as in Eq. (4), and this dose will be independent of the wedge effect. The wedge effect in the dose is recovered using the STT methodology where Y in this case is truncated to the desired field length (in the wedge motion direction) according to the equation:

(14) $$STT_{\theta }(Y)=\left(\frac{w_{\theta }GSTT(Y)+w_{0}GSTT(0)}{w_{\theta }GSTT(Y_{stop})+w_{0}GSTT(0)}\right)$$

In this case, $\text{GSTT}(Y_{\text{stop}})$ is the GSTT at $Y=Y_{\text{stop}}$, where $Y_{\text{stop}}=Y2-0.5$. As described above, STTθ(Y) is converted into 384×512 to constitute a matrix STTθ(i,j). It has been reported that EDW depth dose is almost identical to the open field depth dose;^(^ ^14^ ^–^ ^15^ ^)^ hence, the TPR for open fields can effectively be used in the conversion of EPL to dose. In the presence of an EDW of angle θ, the exit dose in Eq. (4) above is modified to:

(15) $$D_{ext}(i,j)\;\;\;\;=MUxSTT_{\theta }(i,j)xOFxB(s)x\frac{PETD(i,j)}{100}xE_{p}(r,z)xB_{p}(x,z,s)$$

Equation (14) above was evaluated by irradiating, subsequently acquiring EPID images and MapCHECK measurements for approximately 20 cm water‐equivalent thickness (15 cm of solid water plus MapCHECK device (as described in the first paragraph of subsection C above) for 15°, 30°, and 45° EDW of different field sizes. Corresponding plans were generated with the Eclipse TPS for exit dose comparison.

## III. RESULTS & DISCUSSION

### A. Monitor unit effects on equivalent path length

**Figure 2:**
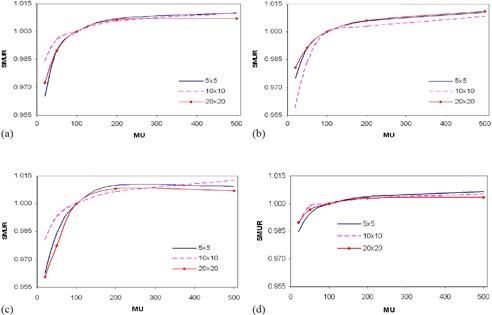
Figures (a), (b), and (c) show SMUR for thicknesses 10 cm, 20 cm and 32 cm measured at 400 MU/min, respectively; (d) shows SMUR for thickness 20 cm measured at 100 MU/min.

(Figures 2a), (b), and (c) show SMUR variations for thicknesses of 10, 20, and 32 cm respectively, measured at 400 MU/min dose rate, and for square field sizes of 5, 10, and 20 cm. The results demonstrate significantly reduced SMUR values at lower MU. (Figure 2d) shows a repeat of SMUR but measured at a reduced dose rate of 100 MU/min for solid water thickness of 20 cm. The SMUR however, increased significantly for 100 MU/min compared to 400 MU/min for low delivered MU. The increase was approximately 3% for MUs below 50. Another notable difference was the relative stability of the SMUR beyond 100 MUs for the lower dose rate, in contrast to the variability measured at 400 MU/min. For the EPID to predict dose effectively, it is desirable for the EPL calculation to be accurate and independent of the delivered number of monitor units. Ideally, the response of any radiation detector should be linearly proportional to MU for both open and MLC blocked beams.^(8)^ The concept of linear relationship suggests that two quantities are directly proportional to each other for all situations, such that the ratios between corresponding entities give the same value. Results for SMUR show a nonlinear relation at MU values below 50.^(^ ^16^ ^–^ ^17^ ^)^ The more consistent SMUR is at a lower dose rate of 100 MU/min than at 400 MU/min indicates that the effect may be due to dead time within the imager system. The SMUR measured at 400 MU/min dose rate was dependent on MU only, but not field size or phantom thickness. The effect can be associated with dead time in EPID frame acquisition, which results in reasonable loss of signal.^(18)^ To eliminate this effect, a single lookup table (MU versus SMUR) was included in the EPL determination.

**Figure 3:**
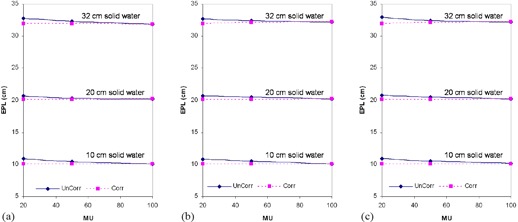
Pre‐MU (UnCorr) and post‐MU (Corr) EPL (calculated before and after MU correction, respectively) for 10 cm, 20 cm, and 32 cm solid water and field sizes of (a) $5\times 5\;{\text{cm}}_{2}$, (b) $10\times 10\;{\text{cm}}^{2}$, and (c) $20\times 20\;{\text{cm}}^{2}$.

The accurate determination of EPL is essential in the prediction of dose by EPID. Figure 3 demonstrates the EPL for different thicknesses of solid water calculated before and after MU corrections, at different field sizes. The results show that at 20 MU, variations in EPL of up to 12 mm can occur between the corrected and uncorrected measurements. Comparison of the pre‐ MU and post‐MU EPL (EPL calculated before and after MU correction, respectively) shows that the latter produces better conformity with expected EPL and was independent of both applied MU and field size. An increase or decrease in EPL of approximately/equal to 10 mm may result in a decrease or increase in percent exit thickness dose of approximately 3%–5%, depending on field size, and hence a discrepancy approximately 3%–5% in the exit dose.^(5)^

### B. Exit dose for irregular fields

As mentioned above, the irradiated field area and resultant phantom scatter are essential for predicting the EPL from EPID responses. The areas calculated from the EPID images for the test cases of (Figs. 1a) and (b) were 323.6 and 246.4 cm^2^, compared to their expected areas of 322.0 and 247.0 cm^2^, respectively. The deviations between EPID predicted and the geometrically expected areas for all images used in the study, including squares and wedged fields, were within ± 1%. Scattered radiation in portal images depends on beam energy, phantom/patient thickness, and field area.^(^ ^19^ ^–^ ^20^ ^)^ Accurate determination of field area, which is one of the paramount factors required in the establishment of phantom/patient scatter correction — along with EPL and dose — is essential. Because there is no simple method to determine the equivalent square for an irregularly shaped field, the most commonly used technique to predict the scatter/output factor correction is the sector‐integration method,^(^ ^8^ ^,^ ^21^ ^)^ where the irregular field is resolved into sectors of circular beams originating at the point of interest in the phantom or patient. However, in the proposed method, the EPID image pixel resolution is less than 1.0 mm and, as a result, the irradiated areas can be calculated to a high degree of accuracy.

**Figure 4:**
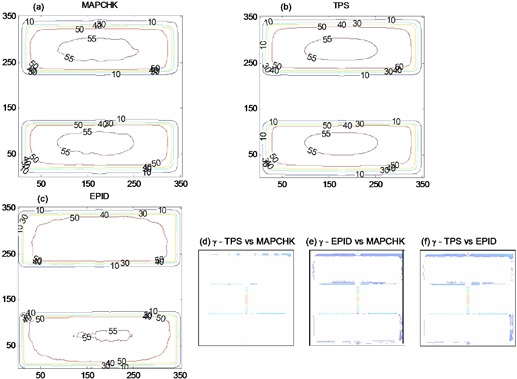
Figures (a), (b), and (c) show 2D absolute exit dose (cGy) distributions measured with MapCHECK device, calculated with TPS and those from EPID images, respectively. Figures (d), (e), and (f) demonstrate the respective 2D gamma maps, evaluated at 3% DD and 3 mm DTA.

**Figure 5:**
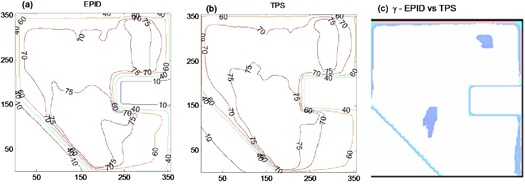
Figures (a) and (b) show EPID and TPS 2D exit dose (cGy) distributions after irradiating an anthropomorphic phantom (Fig. 1(e) with irregular shaped MLC field of Fig. 1(b). Figure (c) demonstrates the corresponding 2D gamma index map.

Figure 4 compares the absolute 2D dose distributions at the exit for the MLC shaped field in Fig. 1(a) as measured by MapCHECK (4(a)), TPS predicted (4(b)), and that calculated from EPID image (4(c)). The percentage of areas where the gamma index ((Figs. 4d), (e) and (f)) evaluated at 3% DD and 3 mm DTA was greater than 1.0, were 3.2%, 8.2%, and 7.6% for TPS vs. MapCHECK, EPID vs. MapCHECK, and TPS vs. EPID, respectively. Figure 5 shows exit dose comparison for the anthropomorphic phantom described in Section II.C, irradiated with Fig. 1(b) field and 200 MU. (Figure 5a) shows the EPID dose distribution calculated from the acquired EPID image of Fig. 1(d), while Fig. 5(b) is the TPS calculated dose distribution. (Figure 5c) shows the corresponding gamma index map, and 91.7% of points passed the 3% DD and 3 mm DTA criteria. The air in the hollow cavity representing sigmoid colon and rectum of the anthropomorphic phantom results in an increased dose along its path, as demonstrated by both the EPID and TPS ((Figs. 5a) and (b), respectively) dose distributions. The MLC shielding of certain areas within a radiation field not only reduces the dose behind the MLC shield, but also reduces the doses in the unshielded areas, in agreement with Boesecke.^(22)^ The EPID, MapCHECK, and TPS calculated doses convey this clearly. Most of the discrepancies are at the MLC defined edges, as illustrated in the 2D dose and gamma index distributions of (Figs. 4d)‐(f) and 5(c). Our utilization of the boundary and envelope correction profiles for open fields may add to this effect. Secondly, the errors at the edges of the MLC might be further minimized by slight adjustment of the field edge of about 1.5–2.0 mm (Varian MLC).^(23)^ The effect this leaf offset might cause on field edge dosimetry needs to be investigated.

### C. Enhanced dynamic fields

**Figure 6:**
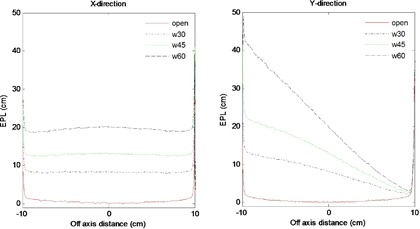
EPL profiles uncorrected for the effects of the wedge in X and Y directions predicted from images obtained without any material between the source and imager for open field (solid lines), 30° (dash‐dot lines), 45° (dot lines) and 60° (dash lines) EDW angles.

Figure 6 shows the EPL cross‐plane profiles for open and EDW fields delivered without any material between the source and EPID detector, at a total delivered MU of 100 and a dose rate of 400 MU/min. The EPL were computed using the set of correction coefficients A(i,j), B(i,j), and $M_{o}(i,j)$ generated for open fields (i.e., uncorrected for the presence of the wedge). For the zero wedge (open) in Fig. 6, the EPL measured across the entire field in the X and Y direction was 0±0.3 cm. For wedged beams, the EPL profiles in the Y (wedge motion) direction are higher at the start of the field and decrease gradually as collimator jaw sweeps across the field. This illustrates the expected effect on delivered dose of the wedge. For the zero‐wedge field, the entire 100 MU is delivered to the entire field, while the EDW comprises the open‐field phase and a collimator‐sweeping phase. From the STT data, it is easily shown that, for a total of 100 MUs, the proportions delivered to the open‐field segments are 53.8 MU, 35.2 MU and 15.6 MU for the 30°, 45°, and 60° EDW, respectively. For the EDW fields, as the total MU increases, the EPL gradually decreases. EDW treatments are optimized such that the intended total MU is delivered by the end of treatment field, explaining why the EPLs in the Y direction are nearly the same. These results clearly depict the inverse proportionality between EPL and MU (dose). The EPL computed for EDW fields using the correction coefficients A(i,j), B(i,j), and $M_{o}(i,j)$ generated for open fields from EPID images is heavily influenced by the motion of the sweeping jaw across the field. Cross‐plane profiles in Fig. 6 illustrate that EPL may rise beyond 20 cm across field for 45° EDW. The limitation with these open‐field coefficients is that, for EDW angles above 30°, the combined EPL contribution from the EDW and irradiated material (~ 20cm solid water) is high, reaching levels beyond the optimized range of calibration values (0–35 cm). Hence, EPL in such cases may be calculated inappropriately. Also, if the combined EPL is greater than the maximum depths for which TPR data is available (40 cm), then the conversion of EPL to dose may also be inaccurate.

**Figure 7:**
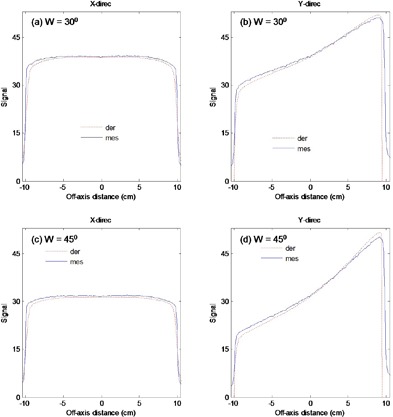
Comparison of measured (solid lines) and derived (dotted lines) profiles without any material in the beam $M_{0}^{\theta }(i,j)$ in the X and Y directions for EDW angles 30° ((a) and (b)) and 45° ((c) and (d)).

Figure 7 shows measured and theoretically derived (Eq. 10) profiles signal without any material $M_{0}^{\theta }(i,j)$ in the beam in the X and Y directions for 30° and 45° EDW angles. The results were for symmetric field size $20\times 20\;{\text{cm}}^{2}(X=20\;\text{cm},Y1=10\;\text{cm}$ and Y2=10 cm). The comparison between measured and calculated profiles shows that the point‐by‐point percentage deviations were less than 1% and 3% in the center and at the fields' edges, respectively.

**Figure 8:**
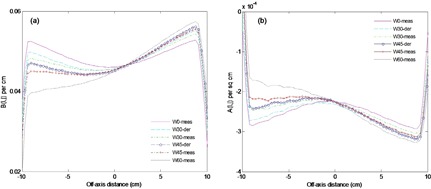
Figures (a) and (b) show the variations of fitting parameter matrices $B_{w\theta }(i,j)$ and $A_{w\theta }(i,j)$ along the Y direction of the EPID respectively for open field (θ=0°),30°,45° and 60° EDW fields. The results also compare the measured and derived profiles for 30° and 45°.

Figures 8(a) and (b) show the variations of fitting parameter matrices $B_{w\theta }(i,j)$ and $A_{w\theta }(i,j)$ along the Y direction of the EPID respectively for open field (θ=0),30°,45° and 60° EDW fields. The results were for a symmetric field size $20\times 20\;{\text{cm}}^{2}(X=20\;\text{cm}$, Y1=10 cm, and Y2=10 cm). The results also compare the measured and derived (Eqs. (11) and (12)) profiles of $B_{w\theta }(i,j)$ and $A_{w\theta }(i,j)$ for the EDW angles 30° and 45°. As illustrated in the results, there are significant variations in $B_{w\theta }(i,j)$ and $A_{w\theta }(i,j)$ for open field and EDW fields. Unlike the open field data, which is symmetric about the central axis, the wedged parameters tend to reflect the slope of the wedge.

**Figure 9:**
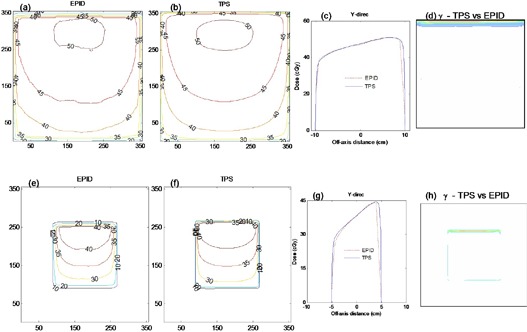
(from L to R) Figures (a) and (b) show absolute 2D exit dose (cGy) distributions for EPD and TPS respectively for a 15° EDW with $20\times 20\;{\text{cm}}^{2}$. Figure (c) shows cross‐plane profiles in the moving jaw direction. Figure (d) shows the 2D gamma map computed from Figs. (a) and (b). Figures (e), (f), (g), and (h) are a repeat of the above for a 45° EDW with $10\times 10\;{\text{cm}}^{2}$ field size.

The theoretically derived data using Eqs. (10) to (13) were used to calculate the exit dose (Eq. (14)) for EDW. (Figures 9a)and (9b) compare the EPID and TPS absolute 2D exit dose (cGy) distribution respectively for a 15° EDW, $20\times 20\;{\text{cm}}^{2}(X=20\;\text{cm}$, Y1=10 cm, Y2=10 cm) field size and delivered MU of 100. (Figures 9c)and (9d) are corresponding cross‐plane profiles in the Y direction and 2D gamma map computed from (Figs. 9a)and (8b), respectively. (Figures 9e), (f), (g), and (h) are similarly 2D exit dose, cross‐plane profiles, and 2D gamma maps and for a 45° EDW, $10\times 10\;{\text{cm}}^{2}(X=10\;\text{cm}$, Y1=5 cm, Y2=5 cm) field size and delivered MU of 100. The profiles in Fig. 9 are practically superimposed within 90% of the irradiated field. The deviations (about 2%) are towards the field edges, as illustrated in (Figs. 9c) and (g). The percentages of areas in (Fig. 9) where the gamma index (3% DD and 3 mm DTA) failed were 4.2 and 5.5 for the 15° and 45° EDW, respectively, which demonstrates a good agreement between the EPID and TPS.

**Figure 10:**
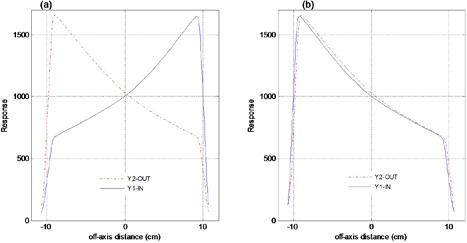
Figure (a) compares EPID image profiles for Y1‐IN (solid lines) and Y2‐OUT (dash lines) for 45° EDW. Figure (b) shows the same data superimposed after taking a mirror image of Y1‐IN data.

All the EDW coefficient data and test cases in this study were acquired with Y1‐IN jaw orientations. Figure 10 compares typical profiles from EPID images acquired for the YI‐IN and Y2‐OUT for 45° EDW. The images were acquired for $20\times 20\;{\text{cm}}^{2}$ with moving jaw Y1=10 cm, fixed jaw Y2=10 cm, and X=20 cm at same SDD and delivered MU of 200. The profiles were extracted from the centers of the images in the EDW motion direction. The results show that EPID pixel value responses for Y1‐IN and Y2‐OUT are practically symmetrical, implying that data for Y2‐OUT jaw orientation can be created from that of Y1‐IN jaw orientation. As expected, the EPID pixel value responses for Y1‐IN and Y2‐OUT for the same radiation field settings are practically symmetrical. This is consistent with the findings of Greer and Barnes^(24)^ Hence the data fitting coefficients for Y2‐OUT can be created from the Y1‐IN by data mirroring, saving valuable re‐measurement time.

**Figure 11:**
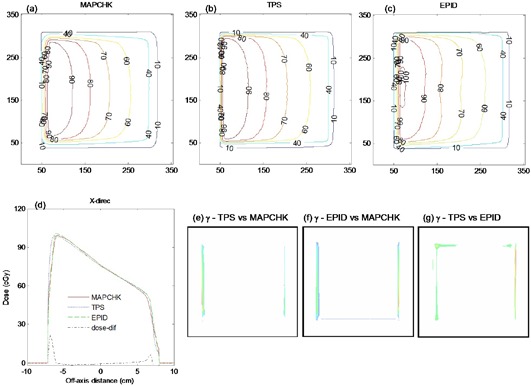
Figures (a), (b), and (c) show 2D absolute exit dose (cGy) distributions measured with MapCHECK device, calculated with TPS and those from EPID images respectively, for a 45°, Y2‐ OUT EDW with $15\times 15\;{\text{cm}}^{2}$. The image was acquired with 200 MU at collimator angle 90°. Figure (d) shows the corresponding exit dose cross‐plane profiles and dose differences between EPID and MapCHECK profiles (dot‐dashed line) in the X direction. Figures (e), (f), and (g) demonstrate the respective 2D gamma maps, evaluated at 3% DD and 3 mm DTA.

Figure 11 compares MapCHECK (11(a)), TPS (11(b)), and EPID image (11(c)) dose distributions, acquired with 45° EDW, $15\times 15\;{\text{cm}}^{2}$ symmetric field size, Y2‐OUT jaw motion, collimator angle 90° and 200 MU. The EPID data used for exit dose calculation were obtained by mirroring the Y1‐IN data, as described above. We used a rotated collimator angle in this example to show that the fitting data coefficients can be used at various beam orientation. The percentages of areas in Fig. 11 where the gamma index (3% DD and 3 mm DTA) failed were 5.5, 7.2, and 7.8 for the TPS vs. MapCHECK, EPID vs. MapCHECK, and TPS vs. EPID, respectively. Reasonably large differences are at the field edges, as illustrated by the EPID vs. MapCHECK dose‐difference profile in Fig. 11(d). However this is not due to using the Y1‐IN data to compute dose for Y2‐OUT, as results in Fig. 9 also show similar effect at the edges.

The deviations at the field edges for EDW fields could be related to observed differences in the $M_{0}^{\theta }(i,j),B_{w\theta }(i,j)$ *and* $A_{w\theta }(i,j)$ data matrices in Figs. 7, 8(a), and 8(b), respectively, that compare cross‐plane profiles of derived and measured coefficients. Towards the edges, both the derived $M_{0}^{\theta }(i,j)$ *and* $B_{w\theta }(i,j)$ are lower than the measured, while $A_{w\theta }(i,j)$ is higher. An interplay between these factors results in an increase in EPL and a decrease in dose towards the field edges. Another possible reason for the reduced accuracy at the edges is the utility of the GSTT described in Eq. (9). It tends to break down at large fields and deviations of 2%–4% between measurement and calculated values have been reported.^(^ ^9^ ^,^ ^10^ ^,^ ^13^ ^)^ The variations in dose rates during EDW dose delivery could be another factor that affecting EPID dose distribution, causing discrepancies. The readout of the a‐Si array are synchronized with the beam pulses, so that EPIDs are calibrated at each accelerator dose rate. The dark and flood field images are different at each dose rate due to variation in image acquisition timing. Dose rate variations that occur during EDW treatments could potentially affect the EPID signal, where the system is calibrated at a fixed accelerator dose rate. Lastly, the disagreements are at the field edges (regions of steep dose gradients) where dose predictions even for the TPS may well be inaccurate.^(24)^ It should be emphasized that for practical in vivo dosimetry, these areas of reduced accuracy at the field edges are much less important. More investigation needs to be done for asymmetric EDW fields. Prado et al.^(9)^ indicated that although simple in approach, the MU fraction model's ability to predict EDW factors accurately is limited for asymmetric fields.

The pixel sensitivity reproducibility of the Varian EPID has been reported to be within 1%.^(^ ^18^ ^,^ ^24^ ^–^ ^26^ ^)^ In a previous study,^(5)^ it is reported that the EPL and fitting coefficients were reproducible to within 1%. This consistency in response gives a high level of confidence in the sensitivity of the system for its intended *in vivo* dosimetry use. Grattan and McGarry^(26)^ investigated the positional stability of the Varian EPID R‐Arm and Exact‐Arm support systems and concluded that the latter, which also is used in this study, provided more reproducible positions than the former. This study indicated that the mean misalignment error for the Exact‐Arm was approximately 2.0 mm, which may not cause adverse effects dosimetrically.

The asymmetry of the portal imager support arm can cause asymmetric backscatter at the imager, which affects the accuracy of dose profiles, particularly in the Y direction^(27)^ and Greer and Barnes^(24)^ reported that backscatter from components of the EPID support arm downstream from the detector can influence the signal by up to 5%. However, in the derivation of the fitting coefficients A(i,j) and B(i,j) (Eq. (1)) in the QCM, the EPID signals M(i,j) obtained after imaging solid water phantoms are divided by the image signal obtained without any material in the beam $M_{0}(i,j)$. Also in Eq. (3), the image signals $M_{1}(i,j)$ whose EPL are to be established are divided with $M_{0}(i,j)$ term. This pixel by pixel division should theoretically eliminate the support arm effect; hence, backscatter correction is not required when using the QCM for EPID dosimetry.^(5)^ Even if the EDW profiles show some consistency with this effect, the errors are not significant compared to those reported by Greer and Barnes.^(24)^

Our exit dose results for open and MLC shield fields can be compared to the work done by Chen et al.^(28)^ This group slightly modified a convolution calibration method that has previously been used to calibrate EPID detector for exit dosimetry. The study limited itself to solid water phantoms less than 11 cm in thickness and an anthropomorphic (head) phantom, in contrast with our study, where we have calculated exit doses for thicknesses in the range of 5–32 cm and a pelvis anthropomorphic phantom. For the centered fields, the EPID profiles in the Chen study fell within 3.1% of the ionization chamber measured dose. This is very much in agreement with our results where, within the irradiated regions, the average dose differences (computed for all cases and for 80% of the irradiated field) between the EPID and TPS is within 3%. Chen et al. observed percent differences ranging from −10% to as much as 65% — which is not much different from what we measured in the MLC shielded regions. This study however, did not include wedged or EDW fields, and we found none in literature to compare with our results.

## IV. CONCLUSIONS

We have shown that the QCM, applied to EPID images of homogeneous solid water phantoms, can be used to predict accurately 2D exit doses for conformal and EDW fields. The inclusion of an MU correction improved the EPL determination and exit dose for various field sizes and thicknesses. The irradiated field areas can be accurately determined from EPID images to within ± 1% uncertainty. Good agreement between the EPID‐predicted, TPS‐calculated, and MapCHECK‐measured dose distributions were obtained for conformal and EDW test cases, with more than 90% of pixels within the irradiated field meeting a gamma index criteria of 3% DD and 3 mm DTA. We conclude that the EPID QCM is an accurate and convenient method for *in vivo* dosimetry and may, therefore, complement existing techniques. Investigation in clinical situations is the subject of our continued work.

## ACKNOWLEDGMENTS

The authors wish to thank the clinical scientists and technical staff at the Beatson Cancer Centre that provided input and discussion for this study. The first author gratefully acknowledges the financial support from the IDB Merit Scholarship Programme.
